# Reverse Tissue Expansion in Gastroschisis: What to do if the Defect is too large to close after Silo Removal?

**Published:** 2014-10-20

**Authors:** Boma T Adikibi, Stuart O'Toole

**Affiliations:** Department of Paediatric Surgery, Royal Hospital for Sick Children, Dalnair street Glasgow, G3 8SJ, UK

**Keywords:** Gastroschisis, Closure, Gore-tex, Delayed infection

## Abstract

A female baby with an antenatal diagnosis of gastroschisis was transferred to our institution. The defect was large but the bowel was in good condition and a silo was placed. After successful reduction of the bowel the abdominal wall defect was too large to allow fascial or even skin closure. We utilised a Gore-tex patch with two prolene purse string sutures placed concentrically to enable the diameter of the patch to be sequentially reduced. This enabled gradual stretching of the tissues with a progressive reduction in the size of the defect. The patch was removed after 8 days and a delayed fascial closure was achieved.

## INTRODUCTION

The management of gastroschisis has evolved in recent times with many institutions utilising a delayed primary closure approach with the use of a silo. We present a case with a large defect where this technique could not be employed and describe an alternative approach.

## CASE REPORT

A female baby was born at 35+1 week gestation by emergency caesarian section due to foetal distress. The diagnosis of gastroschisis was known antenatally. Her birthweight was 2.18 kg which placed her just above the 25th centile. She required minimal resuscitation and was transferred to our institution for further management. 

At arrival, the stomach, small bowel, large bowel, ovaries and fallopian tubes were visible outside to the abdominal cavity. The bowel was matted, but well perfused with no visible atresia or perforation. The defect was to the right of the umbilicus and was large. There was significant abdomino-viscero size discrepancy and primary closure was deemed inappropriate. A size 5 “medicina” silo was placed over the bowel and after a period of 36 hours reduction of the bowel was commenced. 

Reduction of the bowel was difficult and only on day 9 of life was the bowel reduced sufficiently for a plan for later definitive closure. This plan was expedited when turbid fluid was noted around the bowel in the silo, with cellulitis of the surrounding abdominal wall. The baby was pyrexial with an elevated C reactive protein of 122mg/L, cultures taken at the time subsequently grew Burkholderia Cepacia. 

The baby was transferred to theatre for further assessment, the silo removed and the bowel washed with copious amounts of saline. The bowel appeared healthy, but there was still a significant abdomino-viscero size discrepancy. The diameter of the defect was now 5.5cm and fascial closure or even skin closure was not possible. The decision was made to patch the defect using a 1mm gortex patchwith two concentrically placed 3/0 prolene purse string sutures to alow gradual closure of the defect. The patch was secured to the the skin and fasciawith 3.0 prolene horizontal mattress sutures to all layers of the abdominal wall with felt pledglets to protect the skin (Fig. 1).

The baby returned to the neonatal intensive care unit paralysed and ventilated and was extubated after 48hours. The inner prolene purse string was tied on the fourth post operative day reducing the defect by 1.5cm. This was well tolerated and the outer prolene suture was subsequently tied on the sixth post operative day leaving a less than 3cm defect in the abdominal wall (Fig. 2). Two days later, a fascial and skin closure was performed in theatre with removal of the gortex patch (Fig. 3).

Enteral feeding was commenced on day 22; full enteral intake was achieved by day 62 of life. She was discharged on day 70 of life with a good cosmetic appearance of her neo-umbilicus (Fig. 4). She has continued to do well requring no readmission and is thriving.

**Figure:**
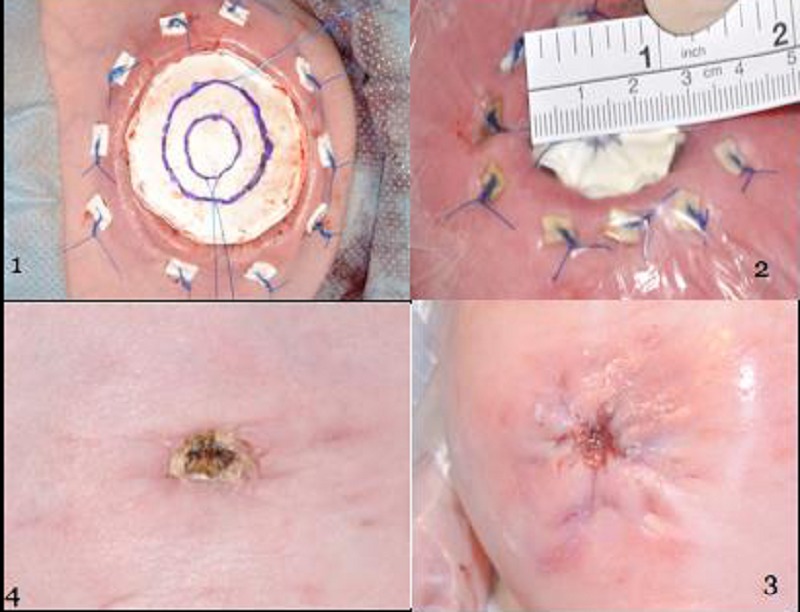
Figure 1-4: 1- The purse string sutures have been placed with care not to include the underlying bowel and the patch has been tied in place with felt pledgets to minimize the trauma to the tissues. 2- Appearance after the tying of both prolene purse string sutures, patch 2.5 cm in diameter. 3- Appearance post fascial and skin closure. 4- Appearance 6 weeks post-surgery.

## DISCUSSION

The management of neonates born with gastroschisis has changed over the past few decades with a shift from primary fascial closure to staged closure with the use of a spring loaded preformed silo. These silos can be placed without anaesthetic at the cotside and delayed fascial closure performed when appropriate [1]. In selected cases, it is even possible to subsequently remove the silo and perform a sutureless closure at the bedside [2]. 

Although the spring loaded preformed silos have many advantages, but we believe that they can make the diameter of the defect larger There have been various methods reported to deal with closure of a large or complex gastroschisis. These include prosthetic materials such as sugisis [3], gortex [4], silastic silo [5] and autologous materials such as umbilical cord [6], dura, musculo-cutaneous flaps and meshed skin grafts [7] reported.

The synthetic agents have the disadvantage of infection causing significant morbidity and necessitating the removal of the patch in some cases. Surgisis has the reported advantage of causing tissue ingrowth and has the theoretical advantage of “growth” but reports have suggested a higher reoccurrence rate with its use. The autologous tissues although intuitively attractive can be problematic in small babies who have a paucity of available tissue.

Our neonate had a very large defect in comparison to the size of her abdomen and the presence of infection precluded the use of other techniques. Gortex was chosen as a temporaizing measure and the concentric purse string suture was devised to reduce the size of the defect for future closure. Sequentially tieing the two sutures at the bedside allowed us to progressively stretch the abdominal wall and close the defect. This progressive tension on the tissues acted like a form of tissue expansion, but in reverse. This technique was performed at the bedside and enabled us to eradicate the sepsis before returning to theatre for definitive closure. Without the use of this innovation, closure would have involved several operative procedures and anaesthetics.

In conclusion, our novel technique for closure of a large gastroschisis defect is easy to perform. It should be considered when the clinician is faced with a defect he cannot obtain skin or fascial closure once he has removed a silo.

## Footnotes

**Source of Support:** Nil

**Conflict of Interest:** None

